# Differential evolution of a CXCR4-using HIV-1 strain in CCR5wt/wt and CCR5∆32/∆32 hosts revealed by longitudinal deep sequencing and phylogenetic reconstruction

**DOI:** 10.1038/srep17607

**Published:** 2015-12-03

**Authors:** Anh Q. Le, Jeremy Taylor, Winnie Dong, Rosemary McCloskey, Conan Woods, Ryan Danroth, Kanna Hayashi, M.-J. Milloy, Art F. Y. Poon, Zabrina L. Brumme

**Affiliations:** 1 Faculty of Health Sciences, Simon Fraser University, Burnaby, Canada; 2 British Columbia Centre for Excellence in HIV/AIDS, Vancouver, Canada; 3 Department of Medicine, University of British Columbia, Vancouver, Canada

## Abstract

Rare individuals homozygous for a naturally-occurring 32 base pair deletion in the CCR5 gene (CCR5∆32/∆32) are resistant to infection by CCR5-using (“R5”) HIV-1 strains but remain susceptible to less common CXCR4-using (“X4”) strains. The evolutionary dynamics of X4 infections however, remain incompletely understood. We identified two individuals, one CCR5wt/wt and one CCR5∆32/∆32, within the Vancouver Injection Drug Users Study who were infected with a genetically similar X4 HIV-1 strain. While early-stage plasma viral loads were comparable in the two individuals (~4.5–5 log_10_ HIV-1 RNA copies/ml), CD4 counts in the CCR5wt/wt individual reached a nadir of <20 CD4 cells/mm^3^ within 17 months but remained >250 cells/mm^3^ in the CCR5∆32/∆32 individual. Ancestral phylogenetic reconstructions using longitudinal envelope-V3 deep sequences suggested that both individuals were infected by a single transmitted/founder (T/F) X4 virus that differed at only one V3 site (codon 24). While substantial within-host HIV-1 V3 diversification was observed in plasma and PBMC in both individuals, the CCR5wt/wt individual’s HIV-1 population gradually reverted from 100% X4 to ~60% R5 over ~4 years whereas the CCR5∆32/∆32 individual’s remained consistently X4. Our observations illuminate early dynamics of X4 HIV-1 infections and underscore the influence of CCR5 genotype on HIV-1 V3 evolution.

Entry of human immunodeficiency virus type-1 (HIV-1) into target cells occurs via binding of the viral envelope protein gp120 to the host CD4 receptor^1^ followed by binding to chemokine coreceptors CCR5 or CXCR4 on the host cell surface^2^,^3^. HIV-1 strains that utilize CCR5 or CXCR4 are termed “R5” and “X4” respectively; those capable of utilizing either coreceptor are termed “R5/X4” (or dual-tropic)^4^. As its principal genetic determinants lie within the third variable (V3) loop of envelope gp120^5^,^6^, HIV-1 coreceptor usage can be determined phenotypically using cell-culture based assays that express patient-derived envelope proteins^7^ or genotypically using algorithms trained on large linked V3 sequence/phenotype datasets^8^,^9^.

R5 strains predominate globally as well as during all infection stages^10^. R5 strains are also preferentially transmitted^10^,^11^,^12^. Before the availability of antiretroviral therapies to treat HIV-1, approximately 50% of individuals who acquired an R5 HIV-1 subtype B strain at transmission would continue to harbor R5 variants throughout their disease course, whereas in the remaining 50%, X4 variants would eventually emerge alongside their R5 counterparts over a timeline of years^13^,^14^,^15^. Referred to as the “coreceptor switch”, this phenomenon is associated with an accelerated clinical progression to AIDS^13^,^14^,^16^, though it remains somewhat unclear whether X4 strains cause, or emerge as a consequence, of immune depletion^17^.

In contrast, acquisition of X4 HIV-1 variants at transmission is less common: recent studies estimate that X4/dual tropic strains comprise between 3–23% of primary infections^18^,^19^,^20^,^21^,^22^,^23^. Though some evidence suggests X4 virus transmission is associated with more rapid clinical progression^24^,^25^,^26^, such infections remain generally less well understood. Moreover, a naturally-occurring 32 base pair deletion in the CCR5 gene (“CCR5∆32”) that results in a non-functional CCR5 protein^27^ also modulates HIV-1 acquisition risk. Specifically, rare individuals homozygous for this deletion (“CCR5∆32/∆32”), who comprise approximately 1% of individuals of European descent^27^,^28^, are resistant to infection by R5 HIV-1 strains but remain susceptible to infection by X4 or dual tropic strains^24^,^27^,^29^,^30^,^31^,^32^,^33^,^34^,^35^,^36^,^37^,^38^,^39^,^40^,^41^,^42^. Resistance to HIV-1 by the CCR5∆32/∆32 genotype is also demonstrated by the “Berlin patient”, the first (and only) individual cured of HIV-1 infection to date, via a stem cell transplant from a histocompatibility-matched donor who additionally carried the CCR5∆32/∆32 mutation^43^.

Major advances have recently been made in our understanding of HIV-1 transmission^11^,^44^,^45^,^46^. We now know that this event is characterized by a severe genetic bottleneck where an estimated 80% of heterosexual infections are productively initiated by a single transmitted/founder (T/F) variant^44^,^46^, whereas infection via injection drug use is generally initiated by more than one closely-related T/F virus^45^. Sequence reconstruction of T/F viruses is traditionally performed by computing a consensus sequence from single-template (*e.g.* clonal, deep-sequenced or single-genome amplified) HIV-1 sequences sampled from plasma shortly after infection^44^,^45^. Alternatively, phylogenetic ancestral reconstruction techniques can been applied to longitudinal single-template HIV-1 sequence datasets–even those sampled weeks or months following infection–to estimate infection dates, reconstruct T/F virus sequences and study within-host HIV-1 evolution in detail^11^,^47^,^48^,^49^. For example, phylogenetic techniques have been applied to longitudinal within-host HIV-1 V3 deep sequence data to reconstruct the timing and emergence of X4 lineages in patients who underwent a coreceptor switch^48^,^50^. In the present study, we apply longitudinal next-generation sequencing and phylogenetic approaches to study a far more rare occurrence: a case where two individuals-one CCR5wt/wt and one CCR5∆32/∆32–were infected with a highly genetically similar X4 HIV-1 strain.

## Methods

### Vancouver Injection Drug Users Study (VIDUS)

Founded in 1996, the original Vancouver Injection Drug Users Study (VIDUS) was a longitudinal cohort comprised of 1603 active injection drug users 18 years or older recruited from the Greater Vancouver area through self-referral and street outreach^51^. At baseline and semi-annual follow-up visits, participants completed a structured interviewer-administered questionnaire and provided a blood sample that was separated into plasma and peripheral blood mononuclear cells (PBMC) and stored at −80 °C until use. PBMC pellets were frozen directly (*i.e.* not cryopreserved); as such, cell separation and viral outgrowth assays were not possible. All individuals completed an HIV test at baseline; HIV-seronegative individuals were tested at each biannual study visit until they seroconverted or until the end of the study. Of 1603 VIDUS participants recruited, 325 (20.3%) were HIV-1 positive (seroprevalent) at study entry whereas 141 (8.8%) seroconverted during follow-up; all other participants did not register an HIV-positive test during follow-up. The present study made use of available bulk plasma HIV-1 RNA and/or DNA sequences spanning Gag, Integrase, V3 and Nef from 115 (of 141, 82.3%) seroconverters and 124 (of 325, 38.2%) seroprevalent VIDUS participants (total 239).

### Ethics statement

Written informed consent was obtained from all participants of the VIDUS cohort. The study was approved by the institutional review boards at Providence Health Care/University of British Columbia and Simon Fraser University, and the study was carried out in accordance with the approved guidelines.

### Amplification and bulk sequencing of HIV-1 RNA and DNA from VIDUS participants

Total nucleic acids were extracted from plasma and PBMC pellets collected from VIDUS participants using standard methods. HIV-1 Gag, Integrase, V3 and Nef were amplified by nested RT-PCR (for HIV-1 RNA) or nested PCR (for HIV-1 DNA) using the Invitrogen SuperScript III One-Step RT-PCR System and/or Roche Expand High Fidelity PCR System respectively, using primers optimized for HIV-1 subtype B. Amplicons were bidirectionally sequenced on a 3130xl or 3730xl automated DNA sequencer (Applied Biosystems). Chromatograms were analyzed using Sequencher v5.0.1 (Genecodes) or custom software RECall^52^ with nucleotide mixtures called if the height of the secondary peak exceeded 25% of the dominant peak height (Sequencher) or 20% of the dominant peak area (RECall). Alignment to the HIV-1 subtype B reference strain HXB2 (for Gag, Integrase and Nef) or a modified subtype B reference sequence (for V3) was done using an in-house alignment tool based on the HyPhy platform^53^. Maximum likelihood phylogenetic trees were constructed using PhyML 3.0^54^. Patristic (tip-to-tip) genetic distances, expressed in terms of substitutions per nucleotide site (sub/nt site), were extracted from maximum-likelihood Newick treefiles using PATRISTIC^55^. Trees were visualized using Figtree v1.4.2 (http://tree.bio.ed.ac.uk/software/figtree/).

### Identification and host genetic characterization of the participant pair

Phylogenetic analysis of population-level bulk HIV-1 sequences identified two VIDUS participants whose HIV-1 sequences exhibited the shortest patristic (tip-to-tip) genetic distances in the cohort, for all HIV-1 genes analyzed (see results). CCR5Δ32 genotyping of these individuals was performed as described previously^56^. Briefly, a ~172 bp region spanning the deletion site was amplified by nested PCR from plasma and/or PBMC-derived DNA and visualized on a 2% agarose gel. To confirm the genotype, 2^nd^ round amplicons were bidirectionally sequenced and chromatograms were visually assessed for length and the presence of the prolonged mixed-base motif characteristic of heterozygous CCR5wt/Δ32 genotypes. In doing so, one individual was identified as homozygous CCR5wt/wt and the other as CCR5Δ32/Δ32. As the latter is a rare genotype, it was confirmed by testing all specimens collected longitudinally from this individual, all with the same result. High resolution HLA class I typing was performed by sequence-based methods^57^.

Clinical estimated dates of infection were calculated as the midpoint between the last HIV-negative and first positive sample, yielding estimates of March 2000 for the CCR5wt/wt individual and August 2001 for the CCR5Δ32/Δ32 individual. As this was a longitudinal study, we arbitrarily designated the CCR5Δ32/Δ32’s estimated infection date as “time-zero” and expressed all other timepoints/specimens relative to this date. For the CCR5wt/wt individual, paired plasma and PBMCs were available at −13, −7, −1, and +35 months, while for the CCR5Δ32/Δ32 individual, plasma samples were available at +5 months, and paired plasma/PBMCs at +6, and +12 months.

The CCR5wt/wt individual, initially antiretroviral naïve, began highly active antiretroviral therapy (HAART) in late August 2001 and remained intermittently on HAART over the course of study followup. The CCR5Δ32/Δ32 individual remained antiretroviral-naive over the course of study followup.

### Longitudinal deep-sequencing of HIV-1 V3 RNA and DNA

Prior to deep-sequencing, the V3 region was re-amplified in triplicate from all plasma and PBMC-derived nucleic acid extracts obtained from each individual. Nested second round amplification was performed using forward and reverse primers incorporating one of 12 multiplex identifier (MID) tags and a linker sequence at the 5′ end and visualized on a 1% agarose gel. Amplicons were quantified with the Quant-iT PicoGreen dsDNA Assay Kit (Invitrogen) on a DTX 880 Multimode Detector (Beckman Coulter), pooled in equal proportions, purified, re-quantified, and deep-sequenced using the GS Junior Titanium Sequencing Kit on a GS Junior instrument (Roche/454). To avoid low-level, intra-run sequence cross-contamination by genetically similar amplicons, we sequenced each sample on a separate GS-Junior run (as each run typically included 24 V3 amplicons, this means that each run contained one amplicon from the present study and 23 V3 amplicons from patients unrelated to the present study). The one exception was sample +35M^Plasma/PBMC^ from the CCR5wt/wt individual, where data are derived from an initial run that included other study samples. Inclusion of data from this sample was deemed appropriate after quality-control experiments confirmed that HIV-1 sequences and their distributions obtained from separate vs. combined runs were highly concordant (not shown).

### Processing of deep sequencing data

Raw sequences were processed, aligned, and trimmed to a modified HIV-1 HXB2 V3 reference standard (HXB2 gp120 codons 296–331) using an iterative process as described previously^58^. Briefly, identical sequences were collapsed and annotated with read counts. Sequences were discarded if the MID or primer sequence was a mismatch to the one assigned to the sample or the sequence did not align to the V3 reference standard. Sequences that were identical except for 1-2 gap characters (attributable to erroneous indels introduced during sequencing) were merged, and read counts updated. The remaining sequences were re-aligned to generate a sample-specific consensus sequence, which was used as the reference standard in subsequent steps.

After realigning all sequences to the specimen-specific consensus, any gap characters followed by ≥3 instances of the same nucleotide were replaced with that nucleotide (to correct for the GS-Junior platform’s difficulty in sequencing homopolymer repeats), and insertions/deletions (indels) were moved to be in-frame. Identical sequences were again merged and read counts updated. A multiple alignment was performed on all remaining sequences, and sequences observed at frequencies of <1% that still contained a single gap character were discarded. To remove any low-level sequence contamination from other patient-derived amplicons sequenced in the same run, an intra-run cross contamination check was performed. To do this, the 5 most frequent sequences within each run (that were observed at a >10% overall prevalence) were identified. Every sequence in our sample was then compared against this list and discarded if it represented an exact match. Lastly, nucleotide sequences with read counts of ≤2, those not divisible by 3 after removal of gap characters, those not encoding cysteines (C) as the starting and final V3 residues, and those <96 or >189 base pairs were discarded as invalid prior to final analysis^59^. Overall, 8 to 55% of raw sequences were discarded as a result of this processing pipeline.

### Ancestral phylogenetic reconstructions

Ancestral phylogenetic reconstructions of intra-host HIV-1 evolution, including the estimation of transmitted/founder (T/F) sequences and dates, were performed using deep sequence data from the three plasma specimens collected closest to “time zero”: −13 M, −7 M, and −1 M for the CCR5wt/wt individual and +5 M, +6 M, and +12 M for the CCR5Δ32/Δ32 individual. To maximize information incorporated into the phylogeny, a ~250 bp sequence encompassing V3 and flanking regions (mapping approximately to HXB2 genomic nucleotides 7086–7336) was used. Processing of V3 deep sequence data for ancestral reconstructions was done using an in-house pipeline described previously^48^. Briefly, raw sequences were grouped by their unique MID tag, and nucleotides with low quality scores (as reported by Roche GS-Junior software) were trimmed from the 5′ and 3′ ends. Identical sequences were temporarily collapsed and annotated with read counts. These were subsequently aligned using a custom sequence alignment algorithm in HyPhy^53^ that adjusts for the high indel rates observed with the GS-Junior platform by aligning all three reading frames to a reference protein standard spanning HXB2 gp120 codons 278–375. This algorithm assumes that a true V3 sequence will encode a single open reading frame, with any frameshifts attributable to erroneous indels introduced during sequencing. Aligned sequences were then re-expanded by their read counts and annotated with sample dates expressed in terms of days elapsed since January 1, 1990.

Time-calibrated phylogenies were reconstructed using Bayesian Evolutionary Analysis Sampling Trees (BEAST) v1.6.1^60^ using parameters described previously with some modifications^48^. Briefly, 100 sequences were randomly sampled from each timepoint, for a total of 600 sequences included in each reconstruction. These 600 sequences were aligned using MUSCLE v3.8.31^61^ and alignments were manually curated using Se-Al (http://tree.bio.ed.ac.uk/software/seal/). Alignments were converted into a BEAST XML file with the following parameter settings: Tamura-Nei^62^ nucleotide substitution model; uncorrelated lognormal molecular clock; Bayesian skyline model with 5 population size classes; and a chain length of 10^8^ with chain states written to log files at intervals of 10^5^ with a burn-in period of 2 × 10^7^ (20%). The resulting trees were then thinned down to 100 sampled at regular intervals. Convergence of chain states was assessed using Gelman and Rubin’s convergence diagnostic implemented in the R package *coda*^63^. For each tree, a Muse-Gaut codon substitution model crossed with a general time-reversible model of nucleotide substitution (implemented in HyPhy^53^) was fit to every tree. Ancestral sequences were generated by sampling 100 character states from the posterior distributions reconstructed at every node of the tree. In total, 10 independent ancestral reconstructions, each randomly sampling 100 sequences per timepoint for a total of 600 sequences, were performed.

### Assessing V3 sequence divergence and diversity

Within-host HIV-1 genetic divergence over time was assessed by calculating patristic (tip-to-tip) phylogenetic distances between each host’s reconstructed T/F virus and all the sequences observed in their plasma and PBMC specimens thereafter, taking into consideration the frequency of each sequence. Within-host HIV-1 diversity, calculated as per-codon differences in Shannon entropy, were calculated from V3 amino acid alignments from the earliest and latest plasma timepoints using Entropy-Two (http://www.hiv.lanl.gov/content/sequence/ENTROPY/entropy.html) using 1000 randomizations with replacement.

### Inference of HIV-1 coreceptor usage

HIV-1 coreceptor usage (R5 vs. X4) was predicted from bulk and deep HIV-1 V3 sequences using geno2pheno_[coreceptor]_ (g2p)^8^. This algorithm assigns each sequence a false-positive rate (FPR) that represents the probability of classifying an R5-virus falsely as X4. We employed a false positive rate (FPR) of 5.0%, meaning that V3 sequences with FPR ≤5.0% and >5.0% were classified as X4 and R5, respectively.

## Results

### Acquisition of a similar X4-using HIV-1 strain in CCR5wt/wt and CCR5∆32/∆32 hosts

**Figure 1:**
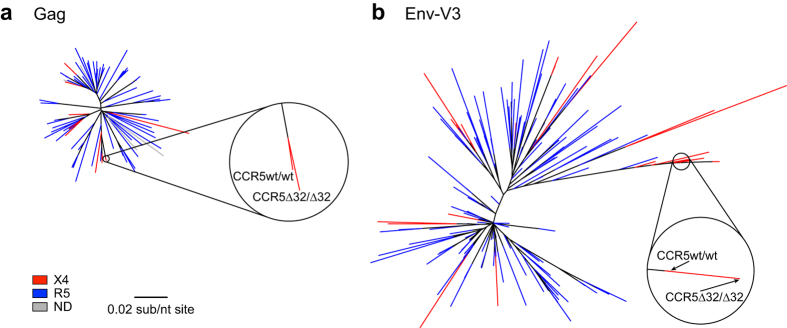
Maximum likelihood phylogenies of bulk HIV-1 Gag and V3 sequences from VIDUS participants. Maximum likelihood phylogenetic trees were constructed using available bulk Gag (*panel **a***) and V3 (*panel **b***) sequences from acute and chronically infected participants of the Vancouver Injection Drug Users Study. The CCR5wt/wt and CCR5∆32/∆32 individuals’ sequences are shown in the zoomed-in window. Tree tips are coloured according to coreceptor usage predicted using V3 genotypes: red for X4-using, blue for R5-using sequences and gray for Gag sequences for which no corresponding V3 sequence was available for coreceptor prediction (ND; not determined).

Maximum-likelihood phylogenies were constructed using one bulk HIV-1 plasma RNA or PBMC DNA Gag, Integrase, V3, and Nef sequence per individual for 239 VIDUS participants (Fig. 1 and Supplementary Figure S1). Computation of patristic (tip-to-tip) genetic distances within these phylogenies consistently identified a participant pair who exhibited the lowest overall distances for all HIV-1 genes examined: these were 0.0027 substitutions per nucleotide site (sub/nt site) in gag (compared to a cohort median of 0.064 [IQR 0.055-0.070]), 0.0023 for integrase (cohort median of 0.034 [IQR 0.025-0.041]), 0.010 for V3 (cohort median of 0.087 [IQR 0.056-0.12]) and 0.023 for nef (cohort median of 0.10 [IQR 0.081-0.11]). The overall prevalence of X4 HIV-1 among all VIDUS seroconverters and seroprevalent participants studied, inferred from bulk V3 sequences, was 13% (12% among seroconverters sequenced within the first year of infection). Over one-third of these resided in a single large cluster that contained the pair of interest (Fig. 1): their bulk V3 sequences were predicted as X4 with g2p FPR values of 1.7% and 2.8%, respectively. CCR5 genotyping further revealed that one individual of the pair was CCR5wt/wt whereas the other was homozygous CCR5∆32/∆32. Neither individual expressed classical “protective” HLA class I alleles^64^: their types were A*03:01/A*31:01, B*07:02/B*51:01, C*07:02/C*14:02 (CCR5wt/wt) and A*23:01/A*25:01, B*35:08/B*44:02, C*04:01/C*05:01 (CCR5∆32/∆32).

**Figure 2:**
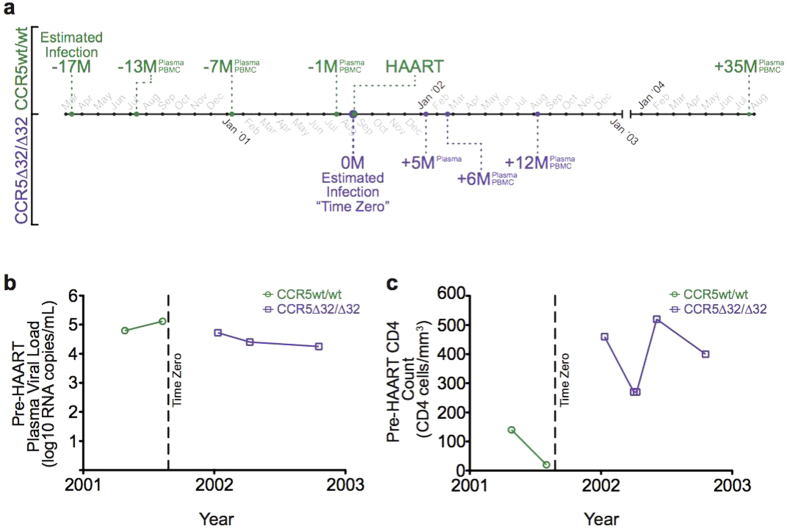
Sampling timeline and clinical histories for the CCR5wt/wt and CCR5∆32/∆32 hosts. *Panel* (***a***): Timelines for the CCR5wt/wt (above, green) and CCR5∆32/∆32 (below, purple) individuals are shown. Putative infection dates (March 2000 for CCR5wt/wt and August 2001 for CCR5∆32/∆32 individuals) were estimated as the midpoint between their last HIV-negative and first HIV-positive tests. The CCR5∆32/∆32 individual’s estimated date of infection was arbitrarily set as “time zero” (0 M); all other timepoints were expressed relative to this date. For the CCR5wt/wt individual plasma and PBMC were available −13, −7, −1, and +35 months relative to time zero; for the CCR5∆32/∆32 individual, plasma samples were available +5, +6, and +12 months and PBMC +6 and +12 months relative to time zero. *Panel* (***b***): Available pre-HAART plasma viral loads (pVL) for CCR5wt/wt (green) and CCR5∆32/∆32 (purple) individuals. Maximum pVL for the CCR5wt/wt and CCR5∆32/∆32 individuals were 5.1 and 4.7 log_10_ RNA copies/mL, respectively. *Panel* (***c***): Available pre-HAART CD4 counts for CCR5wt/wt (green) and CCR5∆32/∆32 (purple) individuals. Nadir CD4 counts for CCR5wt/wt and CCR5∆32/∆32 individuals were 20 and 270 CD4 cells/mm^3^ respectively.

HIV-1 infection dates, estimated as the midpoint between the last HIV-negative and first HIV-positive tests, were March 2000 for the CCR5wt/wt individual (timepoint −17 M, see methods) and August 2001 for the CCR5∆32/∆32 individual (timepoint 0 M) (Fig. 2a). The timing of their respective infections and the observation that their HIV-1 sequences were nearly identical at the bulk level suggest that they could represent a transmission pair, with the CCR5wt/wt the putative donor and the CCR5∆32/∆32 individual the putative recipient. However, confirmation of transmission (*e.g.* via participant contact) was not possible due to the retrospective nature of the analysis and ethics guidelines, and involvement of a third individual or intermediary host cannot be ruled out. Regardless, this represents a rare opportunity to study the evolutionary dynamics of a near-identical HIV-1 strain in individuals with distinct CCR5 genetics.

### Marked differences in nadir CD4 T-cell count

We first analyzed available pre-therapy clinical measurements (Fig. 2b,c). The CCR5wt/wt individual’s highest plasma viral load (pVL), 5.1 log_10_ HIV-1 RNA copies/ml, and nadir CD4 T-cell count, 20 cells/mm^3^, were observed 17 months postinfection. This individual initiated HAART <1 month thereafter. The CCR5∆32/∆32 individual’s highest pVL, observed 4.5 months postinfection, was 4.7 log_10_ HIV-1 RNA copies/ml whereas their nadir CD4 count, observed 9 months postinfection, was 270 CD4 cells/mm^3^. CD4 counts in this individual subsequently rebounded to >400 cells/mm^3^ and this individual remained HAART-naïve throughout follow-up.

### Deep sequencing and ancestral reconstruction

Deep sequencing of the HIV-1 V3 region was performed on all plasma and PBMC samples from both individuals using the Roche GS-Junior Platform. A median of 3143 (range 1905–7248) high quality sequences per sample were analyzed. In addition, 10 phylogenetic ancestral reconstructions were performed using 100 randomly sampled sequences from the three CCR5wt/wt and CCR5∆32/∆32 individuals’ plasma specimens collected closest to “time zero” (the latter’s infection date, see methods). Although the genetic similarity of these infections suggests they may represent a transmission pair, our reconstruction of the evolving lineages on a single timeline is not contingent on this being the case. This reconstruction provides a genetic and temporal context for the evolutionary fate of an unusual virus lineage transmitted from one host to another, possibly through an unknowable number of intermediate hosts.

**Figure 3:**
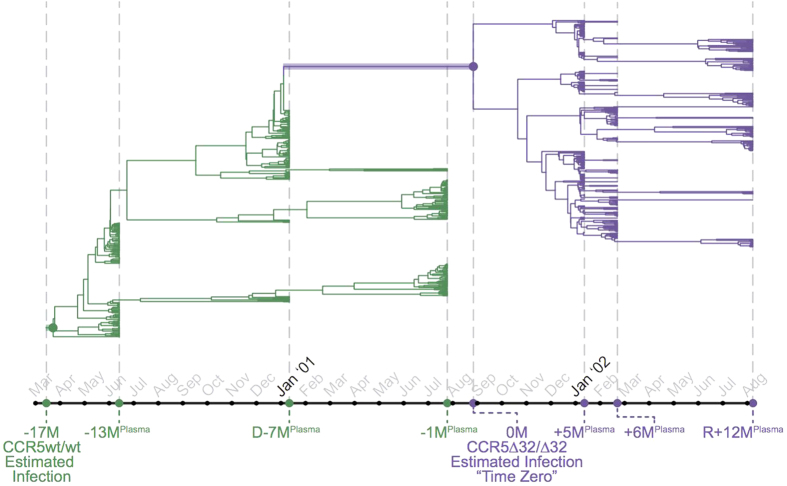
Ancestral phylogenetic reconstruction of HIV-1 V3 transmission/evolution. N = 10 ancestral phylogenetic reconstructions were performed by sampling 100 *plasma* HIV RNA-derived ultradeep sequences per timepoint for the three CCR5wt/wt (green) and three CCR5∆32/∆32 (purple) timepoints closest to time zero. A representative reconstructed phylogeny is shown. Shaded branches and their associated internal nodes represent phylogenetically-inferred date ranges for the CCR5wt/wt (green) and CCR5∆32/∆32 (purple) individuals. This ancestral phylogenetic reconstruction indicates that both CCR5wt/wt and CCR5∆32/∆32 individuals were productively infected by a single X4 virus, within a time period that coincides with the clinical estimated dates of infection. The remaining nine phylogenetic reconstructions were also consistent with transmission of a single T/F virus; in addition, 9 of 10 reconstructions yielded estimated infection date ranges that coincided with clinical estimates. Two additional reconstructions are shown in supplementary figures S2 and S3.

**Figure 4:**
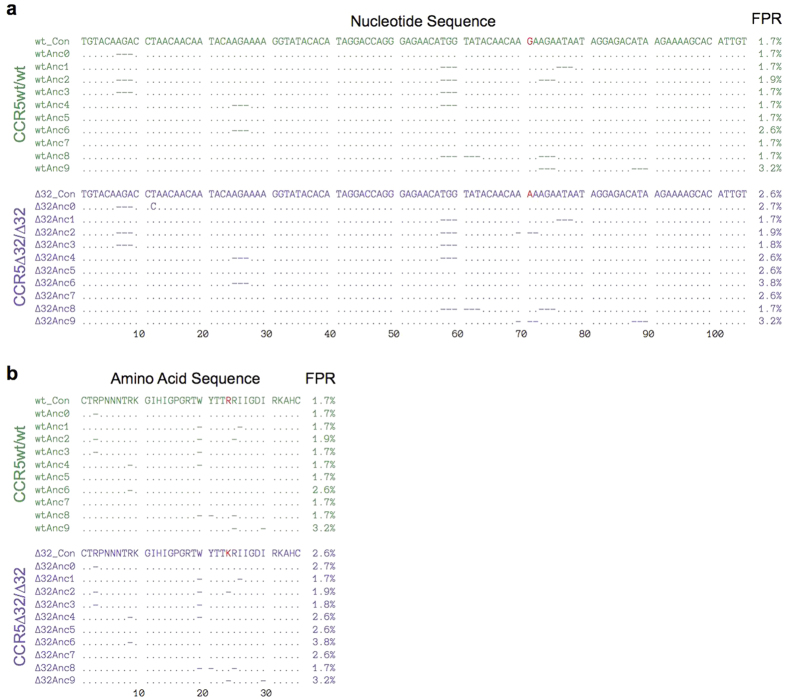
Nucleotide and protein alignments of reconstructed transmitted/founder viruses. Nucleotide and amino acid sequence alignments of the reconstructed T/F virus sequence that infected the CCR5wt/wt (green) and CCR5∆32/∆32 (purple) individuals. The consensus sequence of all 10 ancestral reconstructions (labeled “wt_Con” and “∆32_Con” for the CCR5wt/wt and CCR5∆32/∆32 individuals respectively) is used as the reference. Periods (“ . ”) indicate positions where the sequence is the same as the reference and dashes (“−”) indicate deletions. The “FPR” value following each reconstructed T/F sequence denotes its false-positive rate assigned by geno2pheno_[coreceptor]_^8^; sequences with FPR ≤5.0% are considered X4. *Panel* (***a***): CCR5wt/wt (top, green) and CCR5∆32/∆32 (bottom, purple) nucleotide acid alignments. Consensus nucleotide differences between the CCR5wt/wt and CCR5∆32/∆32 individuals are shown in red. *Panel* (***b***): CCR5wt/wt (top, green) and CCR5∆32/∆32 (bottom, purple) amino acid alignments. The single amino acid difference between the two T/F viruses (at codon 24) is shown in red.

All 10 ancestral reconstructions suggested that HIV-1 infection in both individuals was established by a single T/F viral strain (Fig. 3, Supplementary Figure S2 and S3). Moreover, the phylogenetically-estimated infection time ranges, averaged over all reconstructions (March to April 2000 for the CCR5wt/wt individual, and January to September 2001 for the CCR5∆32/∆32 individual) corroborated the clinically estimated infection dates for these persons. The T/F virus sequences for both individuals (estimated as the consensus of all 10 ancestral reconstructions performed) were predicted as X4 (median g2p FPR 1.7% [range 1.7–3.2%] for the CCR5wt/wt individual and 2.6% [range 1.7–3.8%] for the CCR5∆32/∆32 individual) (Figs 3,4, Supplementary Figure S2, and S3). Their reconstructed T/F V3 sequences differed by only one amino acid at V3 codon 24: the CCR5wt/wt T/F virus harbored arginine (R) whereas the CCR5∆32/∆32 T/F virus harbored lysine (K) (Fig. 4b).

**Figure 5:**
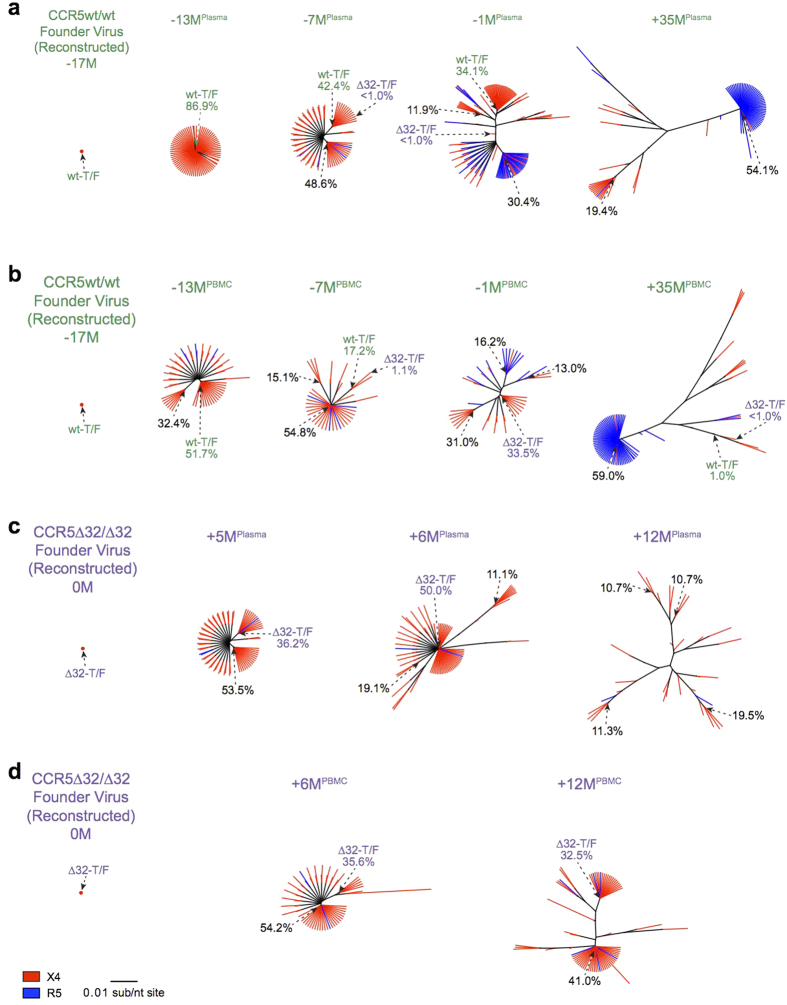
HIV-1 V3 diversification over time. Maximum likelihood phylogenetic trees constructed from unique plasma and PBMC deep V3 sequences from the CCR5wt/wt (panel (**a**) and (**b**) and CCR5∆32/∆32 (panel (**c**) and (**d)**) individuals. Branches are colored by predicted coreceptor usage: red for X4; blue for R5. Prevalent sequences are labeled with their observed frequencies. “wt-T/F” (green) denotes the transmitted/founder virus acquired by the CCR5wt/wt individual; its presence and frequency is tracked throughout the CCR5wt/wt trees. “∆32-T/F” (purple) denotes the transmitted/founder virus acquired by the CCR5∆32/∆32 individual; its presence and frequency is tracked throughout both individuals’ trees. All phylogenies are drawn on the same genetic distance scale.

Reconstruction of T/F virus sequences allowed us to track their frequencies in both hosts over time. After infection, the CCR5wt/wt individual’s T/F virus dominated in plasma (86.9%) at their earliest studied timepoint (−13 M), continued to co-dominate for at least a year thereafter (42.4% at −7 M and 34.1% at −1 M), but was undetectable in plasma at +35 M. Concomitantly, the frequency of this sequence steadily decreased in this individual’s PBMCs, from 51.7% at −13 M, to 17.2% at −7 M and then to low/undetectable levels thereafter (0% at −1 M and 1.0% at +35 M). Similarly, 6 months following infection the CCR5∆32/∆32 individual’s T/F virus sequence remained co-dominant in plasma (36.2% at +5 M; 50.0% at +6 M) but was no longer detected in plasma at +12 M (Fig. 5c,d). By contrast, it remained co-dominant in PBMCs over the entire course of follow-up (35.6% at +6 M and 32.5% at +12 M).

Acknowledging the possibility that the two individuals could represent a transmission pair, we also investigated the frequency of the CCR5∆32/∆32 individual (putative recipient)’s T/F virus sequence in the CCR5wt/wt individual (putative donor)’s plasma and PBMC (Fig. 5a,b). The CCR5∆32/∆32 individual’s T/F sequence was first detectable in the CCR5wt/wt individual at very low frequencies in plasma and PBMCs (<1% and 1.1% respectively) seven months prior to the CCR5∆32/∆32 individual’s estimated infection date. One month prior to the CCR5∆32/∆32 individual’s estimated infection date, the CCR5∆32/∆32 individual’s T/F sequence remained low frequency (<1%) in the CCR5wt/wt individual’s plasma but co-dominated (33.5%) in PBMC. By timepoint +35 M, this sequence was no longer detected in the CCR5wt/wt individual’s plasma and was observed at <1% in PBMC. While detection of this sequence within the CCR5wt/wt individual’s HIV-1 variant pool is intriguing, this observation does not in itself constitute proof of transmission (for example, transmission via one or more intermediary host(s) could have introduced additional unseen genetic bottleneck(s) between our CCR5wt/wt and CCR5∆32/∆32 individuals).

### Divergence from the reconstructed T/F viruses

**Figure 6:**
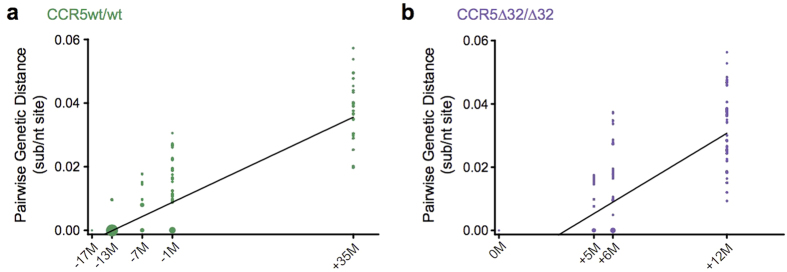
Divergence from the transmitted/founder HIV-1 V3 sequence. *Panel* (***a***) Pairwise genetic distances between the CCR5wt/wt individual’s estimated T/F V3 sequence and all subsequently-observed plasma HIV RNA sequences, measured in substitutions per nucleotide site (sub/nt site). Datapoint sizes reflect observed sequence frequencies, with the largest point representing ~5000 sequences. *Panel* (***b***): Corresponding genetic distances between the CCR5∆32/∆32 individual’s T/F V3 and subsequent plasma V3 sequences.

We next wished to compare the extent to which plasma HIV-1 RNA V3 sequences in both individuals initially diverged from their respective T/F viruses (Fig. 6). To eliminate HAART as a confounder, analysis was restricted to the CCR5wt/wt individual’s first 10 months of infection (pre-HAART period) and a comparable follow-up time for the CCR5∆32/∆32 individual. During this time, the mean divergence from the CCR5wt/wt individual’s T/F virus was 0.0027 sub/nt site, an average rate of divergence of 0.00065 sub/nt site per month. In contrast, mean initial divergence of plasma V3 sequences from the T/F virus in the CCR5∆32/∆32 individual was 0.031 sub/nt site, an average rate of 0.0036 sub/nt site per month, a value that was 5.5-fold higher than that observed in the CCR5wt/wt individual.

### Differential HIV-1 coreceptor usage evolution

**Figure 7:**
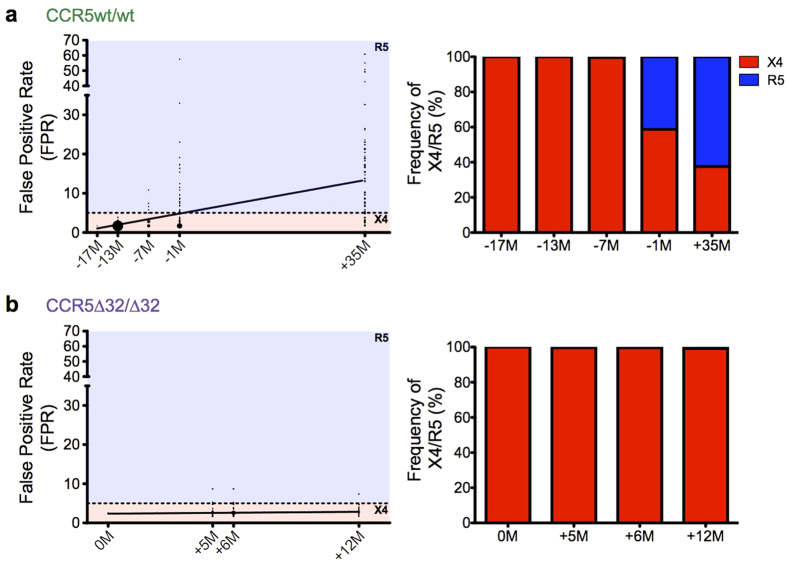
Marked differences in coreceptor usage evolution in CCR5wt/wt vs. CCR5Δ32/Δ32 hosts. *Panel* (***a***) *left:* The false-positive rate (FPR) of HIV-1 coreceptor usage prediction for the CCR5wt/wt individual’s T/F virus (−17 M) and each unique plasma HIV RNA sequence collected thereafter. The horizontal dotted line denotes FPR = 5.0%; sequences with values at or below this threshold are considered X4. *Panel* (***a***) *right:* summarizes the CCR5wt/wt individual’s data in terms of the % of total sequences displaying X4 (red) vs. R5 (blue) usage at each timepoint. *Panel* (***b***): Corresponding analyses for the CCR5∆32/∆32 individual.

We next investigated HIV-1 coreceptor usage evolution in both hosts. In the CCR5wt/wt individual, over a total of 52 months followup, R5 V3 sequences gradually emerged alongside their X4 counterparts (Figs. 5a,b, 7a). The first R5 variants were detected 4 months following infection in PBMC (timepoint −13 M): these early R5 variants comprised <1% of all sequences in this sample and exhibited g2p FPRs in the marginal range (5.1–10.8%). No R5 variants were detected in plasma at this timepoint. By 10 months following infection (timepoint −7 M), R5 variants were detected at <1% frequency in plasma and 1.7% in PBMC, again with marginal FPRs (5.3–10.8%). By 16 months after infection (timepoint –1 M), R5 variants reached frequencies of 41.3% in plasma and 18.1% in PBMC, though FPRs remained marginal (median 8.7% in both compartments). By timepoint +35 M, R5 sequences dominated in both plasma (62.4%) and PBMCs (74.6%), with median FPRs of 18.9% in both compartments (Fig. 7a).

In contrast, in the CCR5∆32/∆32 individual, essentially all (14721 of 14809; 99.4%) plasma and PBMC HIV-1 sequences remained X4 throughout followup (median FPR 2.6% in both compartments) (Figs. 5c,d, 7b). The remaining minority (88 of 14809; 0.6%) of sequences were technically R5, but these exhibited marginal g2p FPRs (range 5.3–8.7%). Moreover, unlike in the CCR5wt/wt individual, the frequencies of sequences with FPRs in this range did not increase over time in the CCR5∆32/∆32 individual.

**Figure 8:**
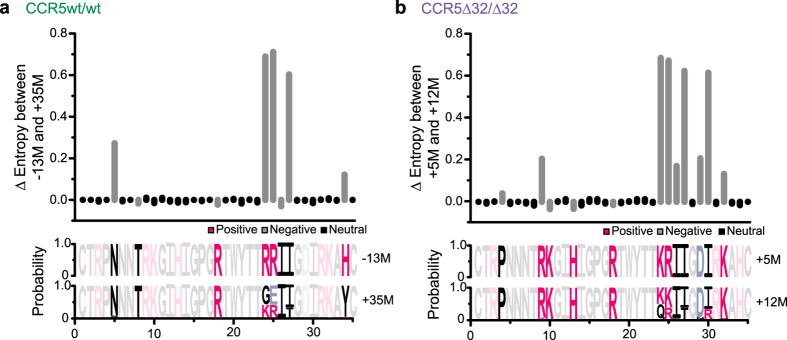
Amino acid diversification at key coreceptor tropism determining sites. *Panel* (***a***) *Top:* Differences in Shannon Entropy (∆ Entropy) between the latest (+35M) and earliest (−13 M) V3 plasma HIV-1 amino acid alignments from the CCR5wt/wt individual. Positive values denote residues that exhibit higher entropy in the later vs. the earlier timepoint (negative values denote the opposite). Significant (p < 0.001) values are shown in Gray. *Panel* (***a***) *bottom*: Corresponding plasma V3 amino acid frequencies at these two timepoints. Positive, negative and neutrally-charged residues are in pink, grey and black respectively, with significantly-changing sites in bright colors and non-significantly-changing sites in dull colors. *Panel* (***b***): Corresponding analysis for the latest (+12 M) vs. earliest (+5 M) plasma V3 sequences from the CCR5∆32/∆32 individual.

We also investigated V3 codon substitutions over time (Fig. 8). For the CCR5wt/wt individual, comparison of the earliest (−13 M) and latest (+35 M) plasma V3 sequences identified five codons (5, 24, 25, 27, and 34) that diversified significantly and three (8, 18, and 26) that contracted modestly during this time (Fig. 8a) (p < 0.001). Codon 25 diversified to the greatest extent, with the X4-associated arginine (R) decreasing from 99.8% to 38.7%. In the CCR5∆32/∆32 individual, comparison of earliest (+5 M) and latest (+12 M) plasma V3 sequences identified nine diversifying (4, 9, 24−27, 29, 30, 32) and three contracting codons (10, 13 and 18) (p < 0.001) (Fig. 8b). Codon 25 also ranked among the most highly diversifying in this individual, with the dominant X4-associated arginine (R) giving way to a 63.5/35.7% mixture of lysine (K)/arginine (R).

## Discussion

We performed longitudinal HIV-1 env-V3 deep sequencing and ancestral phylogenetic reconstruction to study infection (presumably via injection drug use) and subsequent diversification of a highly genetically similar X4 HIV-1 strain in CCR5wt/wt and CCR5∆32/∆32 hosts. A total of 10 independent ancestral reconstructions performed using plasma-derived deep sequences uniformly suggested that both individuals were productively infected with a single, nearly-identical X4 T/F virus, a multiplicity of infection (MOI) which is lower than the reported average for infection via injection drug use (N = 3)^45^. The lower-than-average MOI observed in both individuals is consistent with reports of poorer “transmission fitness” of X4 viruses^11^,^14^,^65^, though larger studies would be required to confirm a relationship between HIV-1 coreceptor usage and MOI in different risk groups. It also argues against the CCR5∆32/∆32 genotype acting as a major additional transmission bottleneck in this case. Consistent with initial rapid diversification of the T/F virus followed by extinction of many viral lineages by immune selection^66^, within-host phylogenies for both individuals were initially starlike but later exhibited a more asymmetrical appearance. Indeed, evidence of selection by neutralizing antibodies was observed in both hosts in the form of diversity loss at V3 codon 18, the final residue of the GPGR “crown motif” within a neutralizing antibody epitope^67^. That this occurred in both hosts is notable as it supports reproducible pathways and timecourse of antibody-driven escape in individuals acquiring genetically-similar viral strains^68^.

Our observations may also shed some insight on the longstanding debate regarding whether X4 viruses cause immune decline (i.e. whether they are inherently more pathogenic than their R5 counterparts), or whether they arise as a consequence of it (i.e. whether R5 and X4 viruses are inherently “fitter” under conditions of relatively preserved immune function versus immunodeficiency, respectively). Our observations indicate that both may be true to some extent. The markedly different nadir CD4 counts observed in the studied individuals supports X4 viruses as inherently more pathogenic when acquired at transmission - but only in CCR5wt/wt hosts. Indeed, this is consistent with previous reports of rapid untreated HIV-1 progression following X4 virus infection in CCR5wt/wt^24^,^25^-but not CCR5∆32/∆32^41^- individuals. On the other hand, the gradual emergence of R5 variants alongside their X4 counterparts in the CCR5wt/wt host over time is consistent with the hypothesis that, under conditions of relatively preserved immune function, R5 viruses have some advantages over their X4 counterparts^12^–possibly in terms of decreased sensitivity to neutralizing antibodies^69^ and/or ability to infect CCR5-expressing CD4 cell subsets^17^,^70^,^71^. Indeed, such X4-to-R5 “reversions” have previously been reported in similar contexts^50^,^72^. Of note, the lengthy timeline and incomplete nature of this process^50^,^72^ suggest that *in vivo* advantages of R5 over X4 viruses are modest in magnitude when the former arise in this context.

Our results also highlight host CCR5 genotype as a major determinant of the evolutionary dynamics of X4 infections. In contrast to the CCR5wt/wt individual who gradually and incompletely reverted to R5, in the CCR5∆32/∆32 individual V3 evolution “explored” genetic space while maintaining a strong X4 phenotype. The CCR5∆32/∆32 genotype did not appear to constrain V3 evolution: initial within-host rates of sequence divergence from the T/F strain in this host were greater than in the CCR5wt/wt individual.

It is not possible to prove the source of HIV-1 transmission using clinical and phylogenetic data alone. Nevertheless, the observation that the two individuals’ patristic (tip-to-tip) HIV-1 genetic distances were the shortest among all VIDUS participants examined (the individual harboring the next closest HIV-1 sequence to the CCR5∆32/∆32 individual exhibited mean genetic distances >5.6-fold [range 1.4–107] greater than those separating the studied pair) and the observation that the CCR5∆32/∆32’s HIV-1 sequences formed a monophyletic lineage nested within a more diverse group of sequences from the CCR5wt/wt individual support the possibility that CCR5wt/wt and CCR5∆32/∆32 individuals represent donor and recipient respectively. Hypothesizing that this is the case, it is interesting to note that at the timepoint closest to the putative transmission event (−1 M), the CCR5∆32/∆32’s inferred T/F strain represented a minority (<1%) variant in the CCR5wt/wt individual’s plasma but co-dominated (33.5%) in PBMC. This in turn raises the intriguing possibility that, if this were a transmission pair, infection of the CCR5∆32/∆32 individual could have occurred via transfer of an infected cell (or via transfer of a minority plasma variant) from the CCR5wt/wt individual. If so, this would corroborate the published finding that T/F viruses cannot be directly predicted from analysis of donor HIV-1 sequences only^73^. Moreover, if the individuals represented a transmission pair the observation that their T/F viruses differed by only one V3 amino acid corroborates the published finding that transmitted viruses tend to be genetically closer to “ancestral” donor viruses than those present in plasma at the time of transmission^49^,^74^. Indeed, the patristic distance separating the individuals’ T/F viruses (0.01704 sub/nt site) was marginally yet significantly lower than that separating the CCR5∆32/∆32 individuals’ T/F virus and plasma viruses present within the CCR5wt/wt individual at the former’s time of infection (0.01711 sub/nt site) (p < 0.0001, Wilcoxon one-sample test).

Several limitations of this study merit mention. First, we infer that HIV-1 infection in both individuals occurred via injection drug use, but sexual transmission cannot be ruled out. Second, as viral templates were not directly quantified (*e.g.* using “primer ID” techniques^75^), possible PCR amplification and/or template resampling biases must be acknowledged, though our approach of triplicate amplifying each extract, quantifying DNA and pooling resulting amplicons equally prior to deep sequencing reduces this concern to some extent^48^. Nevertheless, such biases could conceivably influence our phylogenetic reconstruction of T/F virus sequences, though several lines of evidence indicate this is not the case. Firstly, the composition (in terms of unique sequences and their frequencies) was consistent across plasma and PBMC compartments over time in both hosts. Second, within-host plasma and PBMC phylogenies exhibited characteristic shapes over time. Thirdly, ancestral reconstructions were performed using *plasma* sequences only – but despite this, we detected the reconstructed T/F sequences in PBMCs as well. Finally, replicate sequence data available from 8 of 13 analyzed samples were concordant with the original results in terms of both sequence identity and variant frequencies. Lastly, as this is a descriptive study of a rare event our ability to draw broad conclusions is limited. Nevertheless, our observations highlight the utility of deep sequencing paired with phylogenetic ancestral reconstruction to illuminate early dynamics of X4 HIV-1 infections and underscore the influence of CCR5 genotype on HIV-1 V3 evolution.

## Additional Information

**How to cite this article**: Le, A. Q. *et al.* Differential evolution of a CXCR4-using HIV-1 strain in CCR5wt/wt and CCR5△32/△32 hosts revealed by longitudinal deep sequencing and phylogenetic reconstruction. *Sci. Rep.* **5**, 17607; doi: 10.1038/srep17607 (2015).

## Supplementary Material

Supplementary Information
