# Supplementary material for: Differential evolution of a CXCR4-using HIV-1 strain in CCR5wt/wt and CCR5∆32/∆32 hosts revealed by longitudinal deep sequencing and phylogenetic reconstruction
Source: Sci Rep. 2015 Dec 3;5:17607. doi: 10.1038/srep17607 (PMC4668558; doi:10.1038/srep17607)
Supplement: Supplementary Information [file srep17607-s1.pdf]

**Supplemental Information**

# Longitudinal deep sequencing and phylogenetic reconstruction of CXCR4 HIV-1 transmission to an individual homozygous for the CCR5Δ32 mutation

Anh Q. Le[1], Jeremy Taylor[2], Winnie Dong[2], Rosemary McCloskey[2], Conan Woods[2], Ryan Danroth[1], Kanna Hayashi[2,3], M.-J. Milloy[2,3], Art F.Y. Poon[1,2,3*], Zabrina L. Brumme[1,2*]

1. Faculty of Health Sciences, Simon Fraser University, Burnaby, Canada
2. British Columbia Centre for Excellence in HIV/AIDS, Vancouver, Canada
3. Department of Medicine, University of British Columbia, Vancouver, Canada

* equal contribution

Corresponding Author:
Zabrina L. Brumme
Assistant Professor
Faculty of Health Sciences,
Simon Fraser University
8888 University Drive
Burnaby, BC Canada V5A 1S6
Phone: 778-782-8872
e-mail: zbrumme@sfu.ca

**Supplementary Figure 1**

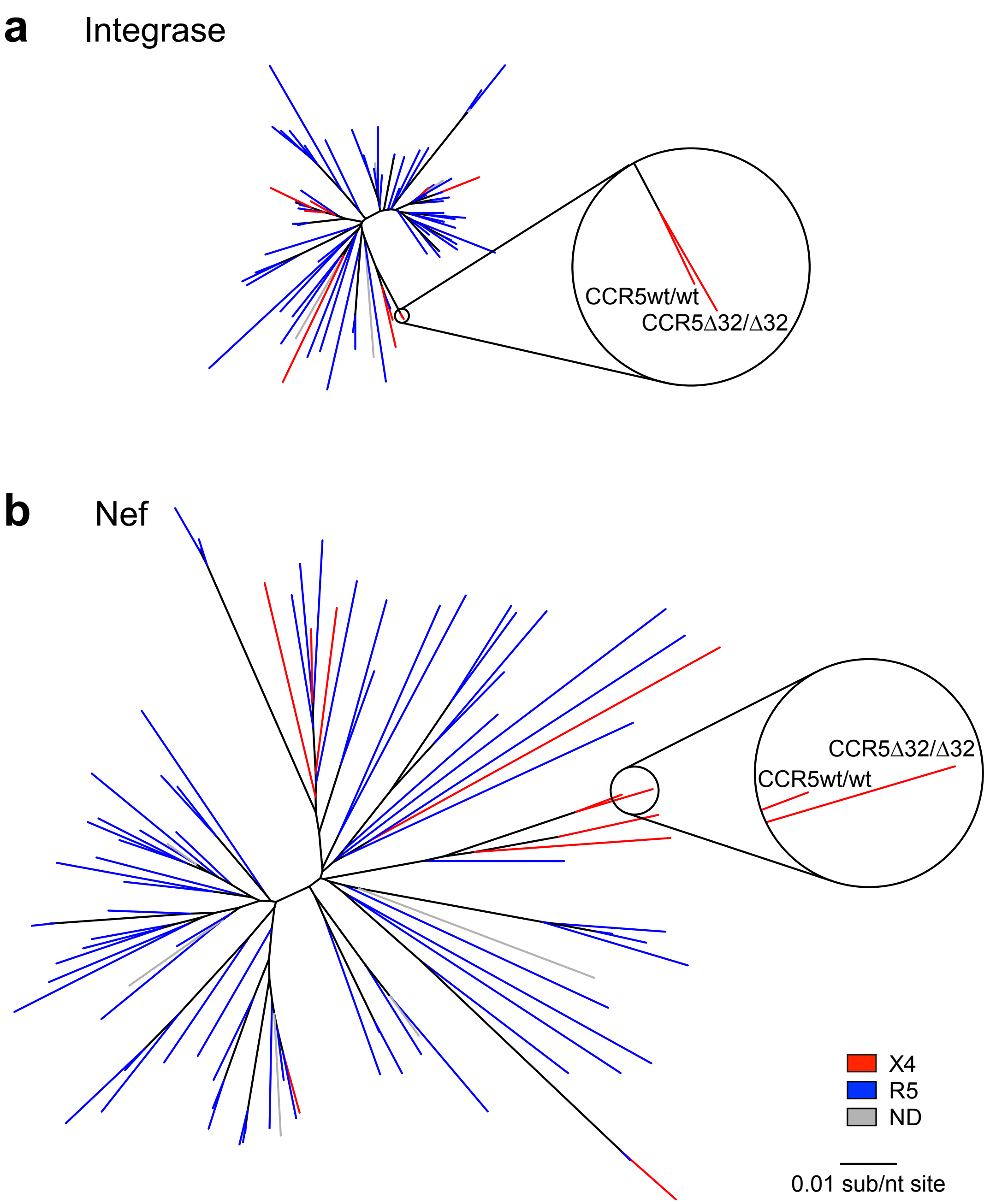


**Figure S1: Maximum likelihood phylogenies of bulk HIV-1 Integrase and Nef sequences from VIDUS participants**

Maximum likelihood phylogenetic trees were constructed using available bulk Integrase (*panel A*) and Nef (*panel B*) sequences from acute and chronically infected participants of

the Vancouver Injection Drug Users Study. The CCR5wt/wt and CCR5Δ32/Δ32 individuals' sequences are shown in the zoomed-in window. Tree tips are coloured according to coreceptor usage predicted using V3 genotypes: red for X4-using, blue for R5-using sequences and gray for Gag sequences for which no corresponding V3 sequence was available for coreceptor prediction (ND; not determined).

**Supplementary Figure 2**

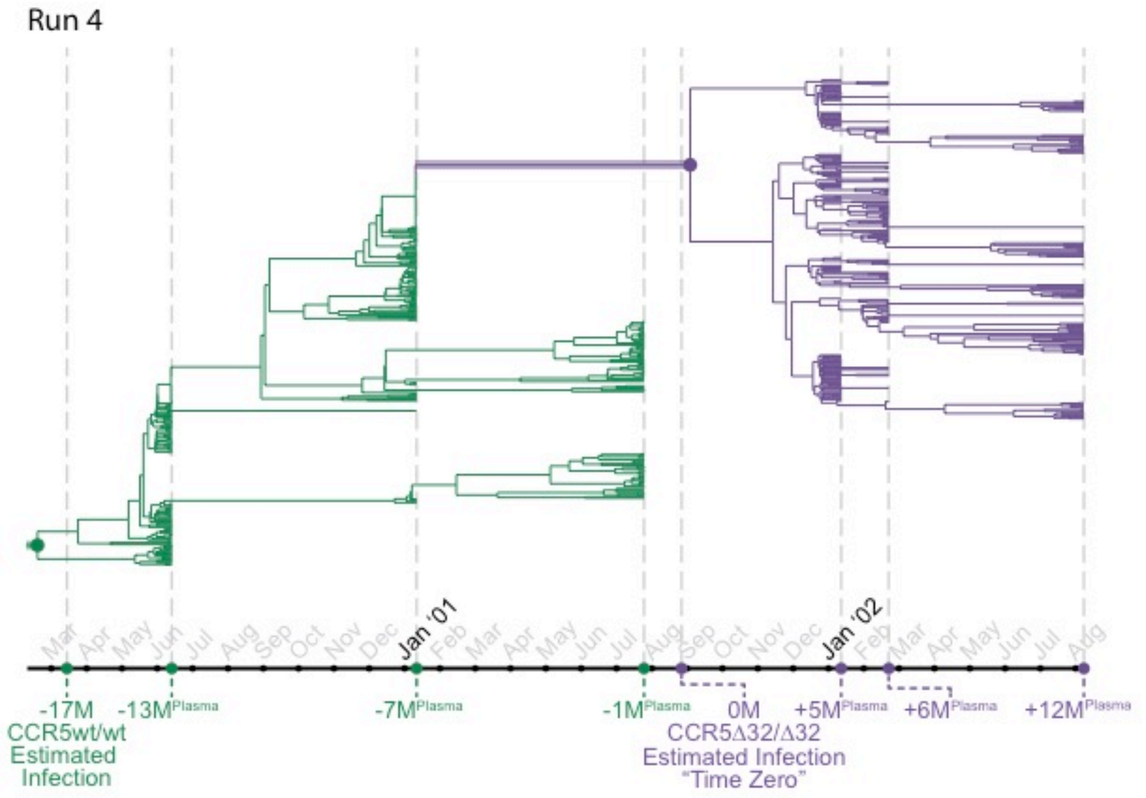


**Figure S2: Second representative ancestral phylogenetic reconstruction of HIV-1 V3 transmission/evolution**

A total of 10 ancestral phylogenetic reconstructions were performed by sampling 100 plasma HIV RNA-derived ultradeep sequences per timepoint for the three CCR5wt/wt (green) and three CCR5Δ32/Δ32 (purple) timepoints closest to time zero. Shown is a second representative ancestral phylogenetic reconstruction. Again, reconstruction supports that the CCR5wt/wt and CCR5Δ32/Δ32 individuals were productively infected by a single X4 virus, within a time period that coincides with the clinical estimated dates of infection (shaded branches).

**Supplementary Figure 3**

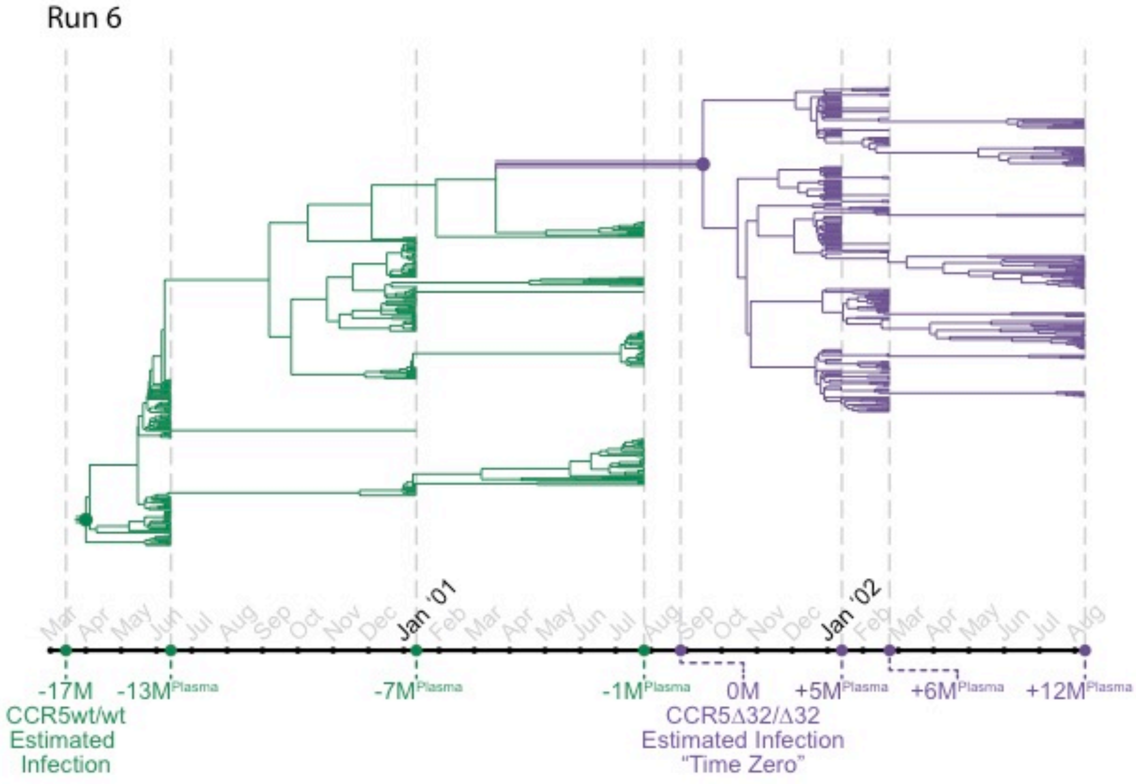


**Figure S3: Third representative ancestral phylogenetic reconstruction of HIV-1 V3 transmission/evolution.** See legend for Figure S2.
